# DNA Methylation as a Therapeutic and Diagnostic Target in Major Depressive Disorder

**DOI:** 10.3389/fnbeh.2022.759052

**Published:** 2022-03-30

**Authors:** Elad Lax

**Affiliations:** Department of Molecular Biology, Ariel University, Ariel, Israel

**Keywords:** epigenetics, DNA methylation, depression, stress, biomarker

Major Depressive Disorder (MDD) is a widespread debilitating neuropsychiatric disorder. While a broad range of drugs to treat MDD are available, a large portion of the patients fail to achieve a complete and sustained remission. It is estimated that only about half of the patients will be responsive to currently available antidepressant treatment (Rush et al., 2006), while others will be only partly responsive, and some will develop a treatment-resistant MDD (Akil et al., 2018).

The etiology of MDD is not clear and considering the large heterogeneity of symptoms and pathophysiologies it is likely to arise from a complex integration of genetic risk factors (Flint and Kendler, 2014; Geschwind and Flint, 2015) and environmental influences, mostly adverse life experiences (Gourion et al., 2008; LeMoult et al., 2020).

Adverse experiences such as early-life stress and poor maternal care are associated with increased risk for MDD in humans (Heim et al., 2010; Lippard and Nemeroff, 2020) and these findings were recapitulated in rodent models (Liu et al., 1997; Caldji et al., 1998) and non-human primates (Champoux et al., 2002; Barr et al., 2003). Maternal behavior and environmental stress alter the development of the hypothalamic–pituitary–adrenal (HPA) axis stress response leading to a stress susceptible phenotype associated with a greater risk for depression later in life (Liu et al., 1997; Anacker et al., 2014). Early-life stress effects can further interact with genetic factors that predispose individuals to depression (Heim and Binder, 2012).

The genome can integrate environmental signals through epigenetic mechanisms such as DNA methylation of CpG dinucleotides and histone modifications. Indeed, ample evidence has linked environmental stress to epigenetic alterations. Increased DNA methylation of the glucocorticoid receptor (GR) promoter was found in the hippocampus of rat pups with poor maternal care (Weaver et al., 2004) and in the post-mortem human brains of individuals who suffered childhood abuse (McGowan et al., 2009). Similar findings suggested conserved epigenetic signature of early life stress in rats and humans (Suderman et al., 2012). It was also demonstrated that peripheral tissues (including blood, saliva and buccal cells) can be used as surrogates for measuring epigenetic changes in the brain across many neuropsychiatric disorders (Fuchikami et al., 2011; Unternaehrer et al., 2012; Lax et al., 2018; McEwen et al., 2020). In addition, many CpGs show correlation of DNA methylation levels between blood and several brain regions, and hence can serve as disease biomarkers (Hannon et al., 2015; Edgar et al., 2017).

The observations that environmental factors, such as early-life stress, that make individuals prone to MDD, also modulate the epigenetic signals to ultimately reprogram brain gene-expression patterns encouraged studies that seek direct associations between MDD and DNA methylation. For example, a genome-wide DNA methylation study in post-mortem brain samples from MDD patients who died during a depressive episode and matched controls found more than a hundred differentially methylated regions between the groups (Nagy et al., 2015). Recently, a large-scale genome-wide study directly compared brain and blood DNA methylation patterns in MDD patients including replication cohorts and found differentially methylated sites in MDD patients (Aberg et al., 2020). Other researchers used a candidate gene approach and found changes in DNA methylation levels for the genes *MAOA* (encoding the monoamine-oxidase-A enzyme) and *NR3C1* (encoding the glucocorticoid-receptor) in individuals with MDD and childhood adversities (Melas et al., 2013).

Other studies aimed to assess DNA methylation levels of the promoter of *SLC6A4*, the gene that encodes the serotonin transporter, a major target of many antidepressant drugs. Kang et al. (2013) found an association between childhood adversity and worse clinical presentation of MDD and higher methylation levels of the *SLC6A4* promoter with no effect of antidepressant treatment on methylation levels of this region. Using the same approach, Okada et al. (2014) did not find a significant difference between DNA methylation levels of the *SLC6A4* promoter in healthy controls and MDD patients before antidepressant treatment. However, they found significantly increased methylation in some CpGs following a 6-week treatment. Similar findings were also found in additional studies (Vijayendran et al., 2012; Zhao et al., 2013; Domschke et al., 2014). Furthermore, several studies linked peripheral measures of *SLC6A4* promoter DNA methylation to brain connectivity in MDD (Chiarella et al., 2020), brain functions involved in emotional stimuli (Frodl et al., 2015), and hippocampal volume in MDD (Booij et al., 2015). Notably, heterogeneity in DNA methylation changes in MDD across experiments is to be expected due to factors such as genomic heterogeneity and the parameters of the sampled population. For example, a distinct DNA methylation signature was found for adult-onset and late-onset MDD (Yamagata et al., 2021). On the other hand, parameters such as ethnicity might have smaller effects. A meta-analysis of multiethnic epigenome-wide studies for depressive symptoms found DNA methylation signatures of depression which were robust across ethnicities (Story Jovanova et al., 2018).

Taken together, the findings that DNA methylation changes were observed in MDD led to efforts to pharmacologically manipulate DNA methylation levels as a potential antidepressant treatment. Administration of S-adenosyl methionine (SAM), a methyl donor that is used by DNA methyltransferases (DNMTs) to catalyze DNA methylation, can increase global DNA methylation. Therefore, many studies examined the effect of SAM administration as a monotherapy or an add-on to antidepressant treatment. The overall effects of SAM administration in MDD were analyzed in several thorough systemic reviews, which concluded that SAM shows promising results although additional larger randomized double-blind studies with long-term follow-up are required (Galizia et al., 2016; Sarris et al., 2016; Sharma et al., 2017; Cuomo et al., 2020). Animal models suggested some mechanistic insight into the beneficial effects of SAM. Saunderson et al. (2016) demonstrated that SAM administration attenuated stress-induced c-Fos and Egr-1 gene-promoter demethylation and protein expression in the dentate gyrus of stressed rats. Intracerebroventricular infusion of methionine (SAM precursor) reversed stress response and DNA methylation levels of the GR promoter in rat offspring from poor maternal care dams (Weaver et al., 2005) and systemic methionine injections in the same animal model altered gene-expression of over 300 genes in the hippocampus (Weaver et al., 2006). Notably, *Dnmt3a* over-expression (which increases global methylation) specifically in the nucleus accumbens increased depressive-like behaviors, while DNMT inhibition with RG-108 decreased depressive-like behaviors in mice (LaPlant et al., 2010). Also, forebrain deletion of *Dnmt1*, but not *Dnmt3a*, showed anti-depressive effects (Morris et al., 2016).

In naïve newborn and adult rodents, systematic administration of the DNMT inhibitors 5-aza-2-deoxycytidine or 5-azacytidine reduced depressive-like behaviors through demethylation of the *Bdnf* gene promoter leading to increased brain Bdnf mRNA and protein levels (Sales et al., 2011; Li et al., 2017). While the findings of pharmacological inhibition of DNA methylation might be seen as contradictory to the beneficial effects of SAM observed in preclinical and clinical studies, it is important to note that different models, species, and administration routes were used, making it hard to directly compare these results. Also, while SAM treatment is a promising add-on MDD therapy, it increases DNA methylation globally and can potentially reprogram gene-expression beyond those that are causative for MDD. However, many human studies on the effects of SAM administration on MDD showed beneficial effects for this treatment with no major adverse side-effects reported. Currently, there are not pharmacological interventions that can manipulate DNA methylation levels of specific genomic loci. Novel technologies might allow this in the future, for example by targeting a catalytically inactive CRISPR/deadCAS9 protein fused to DNMT3a (dCAS9-DNMT3a) to loci of interest as was shown experimentally (Liu et al., 2016; Vojta et al., 2016; Xu and Heller, 2019).

Disentangling whether changes in DNA methylation are the cause or result of MMD is very difficult in human studies which can measure mostly associations. However, several studies found that DNA methylation can predict behavioral outcomes, including major depression, in humans (Ursini et al., 2011; Guintivano et al., 2014; Humphreys et al., 2019). In addition, studies in animal models for MDD showed that manipulating the methyl donor availability, DNMTs levels and DNMTs activity can induce MDD-like behavioral phenotypes (LaPlant et al., 2010; Sales et al., 2011; Morris et al., 2016; Li et al., 2017). Therefore, it is prudent to assume that alterations in DNA methylation, as a result to external stressful stimuli, are at least partly causative of MDD, although it is likely that some methylation alterations are secondary to MDD (and yet can serve as potential biomarkers).

The notion that manipulating DNA methylation has an impact on MDD encouraged studies measuring DNA methylation levels as potential biomarkers to predict MDD and its severity in vulnerable populations as well as treatment outcomes. For example, blood methylation levels were measured in several cohorts to successfully predict antenatal and postpartum depression (Guintivano et al., 2014; Payne et al., 2020). Some of these studies focused on one or a few candidate genes as potential MDD predictors, mostly the *BDNF* and *SLC6A4* genes (Booij et al., 2015; Kleimann et al., 2015), while others used genome-wide methods (Barbu et al., 2020).

A recent systematic review on DNA methylation in depression and the effects of MDD treatment on DNA methylation concluded that findings from studies that aimed to search for biomarkers for MDD treatment outcome are inconsistent; with some studies showing significant results while others had mixed findings. This is most likely due to larger heterogeneity compared to other studies, types and stages of treatment and small sample sizes in some of the studies. Overall, the most consistent effects were increased methylation of the *BDNF* and *SLC6A4* genes in MDD patients (Li et al., 2019).

Taken as a whole, a growing body of evidence support a role for DNA methylation in MDD (see summary in Figure 1). Drugs that modify DNA methylation are available and demonstrate significant effects across both preclinical and clinical studies. These drugs (mostly methionine and SAM) have the potential to be used as adjuvants increasing the efficacy of classic antidepressant treatments. Further, peripheral DNA methylation has the potential to become a non-invasive method for assessing MDD risk and treatment-efficacy estimation. Future large-scale research on MDD patients is needed for further study and validation to establish these approaches.

**Figure 1:**
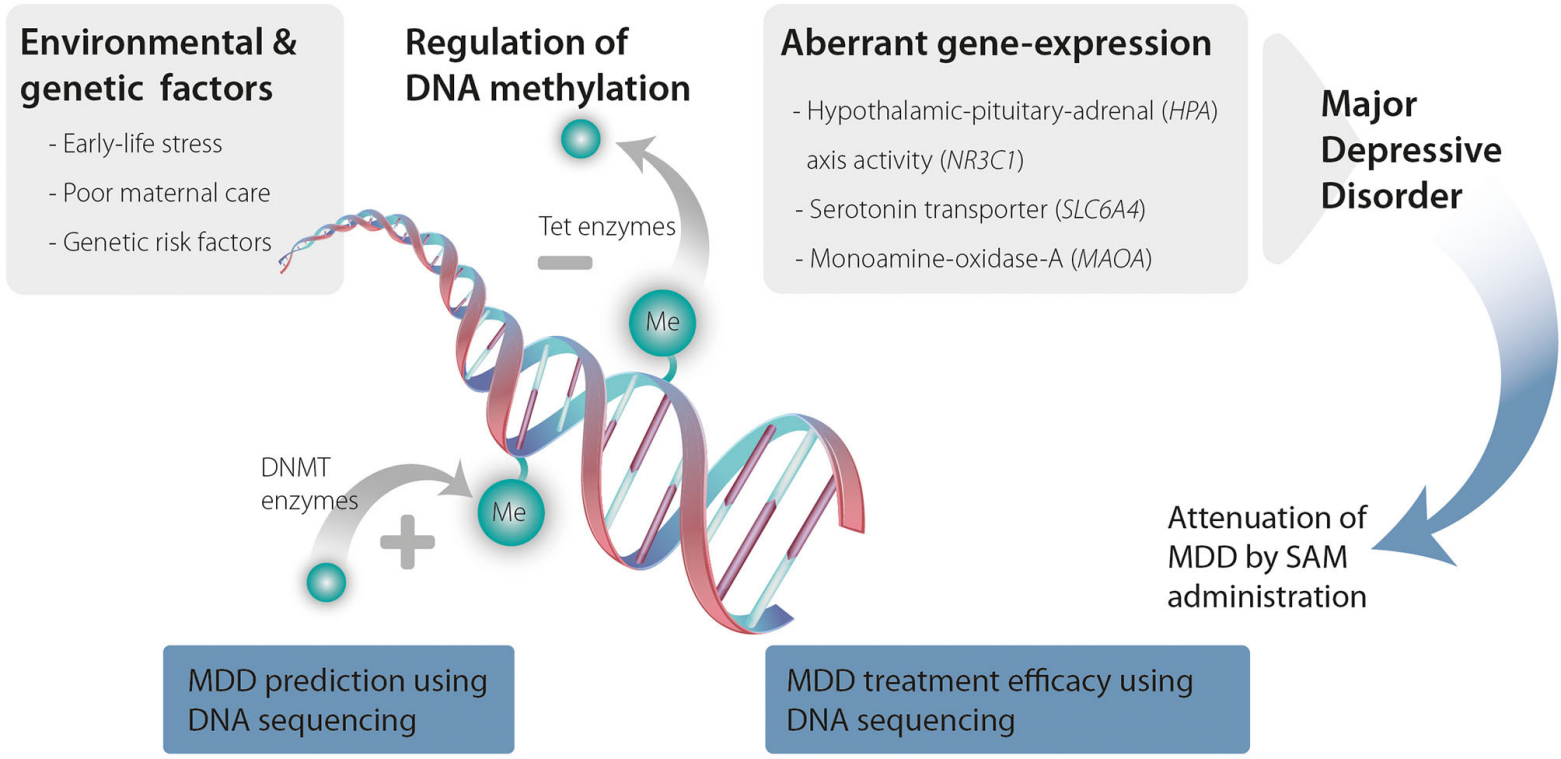
Environmental and genetic risk factors promote changes in DNA methyltransferases (DNMTs) and ten-eleven translocation methylcytosine dioxygenases (TET) activity that increase DNA methylation and demethylation, respectively. Changes in DNA methylation lead to aberrant expression of genes that are associated with MDD ultimately causing MDD. SAM administration can attenuate these changes in DNA methylation. Further, genomic sequencing to detect changes in DNA methylation can serve as a predictive tool for MDD as well as a tool to predict treatment efficacy and responsiveness.

## Author Contributions

The author confirms being the sole contributor of this work and has approved it for publication.

## Funding

This research in EL lab is supported by the Estates Committee of the Israeli Ministry of Justice.

## Conflict of Interest

The author declares that the research was conducted in the absence of any commercial or financial relationships that could be construed as a potential conflict of interest.

## Publisher's Note

All claims expressed in this article are solely those of the authors and do not necessarily represent those of their affiliated organizations, or those of the publisher, the editors and the reviewers. Any product that may be evaluated in this article, or claim that may be made by its manufacturer, is not guaranteed or endorsed by the publisher.

## References

[B1] Aberg K. A. Dean B. Shabalin A. A. Chan R. F. Han L. K. M. Zhao M.. (2020). Methylome-wide association findings for major depressive disorder overlap in blood and brain and replicate in independent brain samples. Mol. Psychiatry 25, 1344–1354. 10.1038/s41380-018-0247-6 30242228 PMC6428621

[B2] Akil H. Gordon J. Hen R. Javitch J. Mayberg H. McEwen B.. (2018). Treatment resistant depression: a multi-scale, systems biology approach. Neurosci. Biobehav. Rev. 84, 272–288. 10.1016/j.neubiorev.08.019 28859997 PMC5729118

[B3] Anacker C. O'Donnell K. J. Meaney M. J. (2014). Early life adversity and the epigenetic programming of hypothalamic-pituitary-adrenal function. Dialogues Clin. Neurosci. 16, 321–333. 10.31887/DCNS.16.3/canacker 25364283 PMC4214175

[B4] Barbu M. C. Shen X. Walker R. M. Howard D. M. Evans K. L. Whalley H. C. (2020). Epigenetic prediction of major depressive disorder. Mol. Psychiatry 26, 5112–5123. 10.1101/19001123 32523041 PMC8589651

[B5] Barr C. S. Newman T. K. Becker M. L. Parker C. C. Champoux M. Lesch K. P. (2003). The utility of the non-human primate; model for studying gene by environment interactions in behavioral research. Genes Brain Behav. 2, 336–340. 10.1046/j.1601-2003.00051.x 14653305

[B6] Booij L. Szyf M. Carballedo A. Frey E. M. Morris D. Dymov S. (2015). DNA methylation of the serotonin transporter gene in peripheral cells and stress-related changes in hippocampal volume: a study in depressed patients and healthy controls. PLoS ONE 10, e0119061. 10.1371/journal.pone.0119061 25781010 PMC4363605

[B7] Caldji C. Tannenbaum B. Sharma S. Francis D. Plotsky P. M. Meaney M. J. (1998). Maternal care during infancy regulates the development of neural systems mediating the expression of fearfulness in the rat. Proc. Natl. Acad. Sci. U.S.A. 95, 5335–5340. 10.1073/pnas.95.9.5335 9560276 PMC20261

[B8] Champoux M. Bennett A. Shannon C. Higley J. D. Lesch K. P. Suomi S. J. (2002). Serotonin transporter gene polymorphism, differential early rearing, and behavior in rhesus monkey neonates. Mol. Psychiatry 7, 1058–1063. 10.1038/sj.mp.4001157 12476320

[B9] Chiarella J. Schumann L. Pomares F. B. Frodl T. Tozzi L. Nemoda Z. (2020). DNA methylation differences in stress-related genes, functional connectivity and gray matter volume in depressed and healthy adolescents. J. Affect. Disord. 271, 160–168. 10.1016/j.jad.2020.03.062. 32479312

[B10] Cuomo A. Beccarini Crescenzi B. Bolognesi S. Goracci A. Koukouna D. Rossi R. (2020). S-Adenosylmethionine (SAMe) in major depressive disorder (MDD): a clinician-oriented systematic review. Ann. Gen. Psychiatry 19, 50. 10.1186/s12991-020-00298-z 32939220 PMC7487540

[B11] Domschke K. Tidow N. Schwarte K. Deckert J. Lesch K. P. Arolt V. (2014). Serotonin transporter gene hypomethylation predicts impaired antidepressant treatment response. Int. J. Neuropsychopharmacol. 17, 1167–1176. 10.1017/S146114571400039X 24679990

[B12] Edgar R. D. Jones M. J. Meaney M. J. Turecki G. Kobor M. S. (2017). BECon: a tool for interpreting DNA methylation findings from blood in the context of brain. Transl. Psychiatry 7, e1187. 10.1038/tp.2017.171 28763057 PMC5611738

[B13] Flint J. Kendler K. S. (2014). The genetics of major depression. Neuron 81, 484–503. 10.1016/j.neuron.01.027 24507187 PMC3919201

[B14] Frodl T. Szyf M. Carballedo A. Ly V. Dymov S. Vaisheva F. (2015). DNA methylation of the serotonin transporter gene (SLC6A4) is associated with brain function involved in processing emotional stimuli. J. Psychiatry Neurosci. 40, 296–305. 10.1503/jpn.140180 25825812 PMC4543092

[B15] Fuchikami M. Morinobu S. Segawa M. Okamoto Y. Yamawaki S. Ozaki N. (2011). DNA methylation profiles of the brain-derived neurotrophic factor (BDNF) gene as a potent diagnostic biomarker in major depression. PLoS ONE 6, e23881. 10.1371/journal.pone.0023881 21912609 PMC3166055

[B16] Galizia I. Oldani L. Macritchie K. Amari E. Dougall D. Jones T. N. (2016). S-adenosyl methionine (SAMe) for depression in adults. Cochrane Database Syst. Rev. 10, CD011286. 10.1002/14651858.CD011286.pub2 27727432 PMC6457972

[B17] Geschwind D. H. Flint J. (2015). Genetics and genomics of psychiatric disease. Science 349, 1489–1494. 10.1126/science.aaa8954 26404826 PMC4694563

[B18] Gourion D. Arseneault L. Vitaro F. Brezo J. Turecki G. Tremblay R. E. (2008). Early environment and major depression in young adults: a longitudinal study. Psychiatry Res. 161, 170–176. 10.1016/j.psychres.07.026 18849082

[B19] Guintivano J. Arad M. Gould T. D. Payne J. L. Kaminsky Z. A. (2014). Antenatal prediction of postpartum depression with blood DNA methylation biomarkers. Mol. Psychiatry 19, 560–567. 10.1038/mp.2013.62 23689534 PMC7039252

[B20] Hannon E. Lunnon K. Schalkwyk L. Mill J. (2015). Interindividual methylomic variation across blood, cortex, and cerebellum: implications for epigenetic studies of neurological and neuropsychiatric phenotypes. Epigenetics 10, 1024–1032. 10.1080/15592015.1100786 26457534 PMC4844197

[B21] Heim C. Binder E. B. (2012). Current research trends in early life stress and depression: review of human studies on sensitive periods, gene-environment interactions, and epigenetics. Exp. Neurol. 233, 102–111. 10.1016/j.expneurol.10.032 22101006

[B22] Heim C. Shugart M. Craighead W. E. Nemeroff C. B. (2010). Neurobiological and psychiatric consequences of child abuse and neglect. Dev. Psychobiol. 52, 671–690. 10.1002/dev.20494 20882586

[B23] Humphreys K. L. Moore S. R. Davis E. G. MacIsaac J. L. Lin D. T. S. Kobor M. S.. (2019). DNA methylation of HPA-axis genes and the onset of major depressive disorder in adolescent girls: a prospective analysis. Transl. Psychiatry. 9, 245. 10.1038/s41398-019-0582-7 31582756 PMC6776528

[B24] Kang H. J. Kim J. M. Stewart R. Kim S. Y. Bae K. Y. Kim S. W. (2013). Association of SLC6A4 methylation with early adversity, characteristics and outcomes in depression. Prog. Neuropsychopharmacol. Biol. Psychiatry 44, 23–28. 10.1016/j.pnpbp.2013.01.006. 23333376

[B25] Kleimann A. Kotsiari A. Sperling W. Groschl M. Heberlein A. Kahl K. G. (2015). BDNF serum levels and promoter methylation of BDNF exon I, IV and VI in depressed patients receiving electroconvulsive therapy. J Neural Transm (Vienna). 122, 925–928. 10.1007/s00702-014-1336-6 25387785

[B26] LaPlant Q. Vialou V. Covington H. E. I. I. I. Dumitriu D. Feng J. Warren B. L.. (2010). Dnmt3a regulates emotional behavior and spine plasticity in the nucleus accumbens. Nat. Neurosci. 13, 1137–1143. 10.1038/nn.2619 20729844 PMC2928863

[B27] Lax E. Warhaftig G. Ohana D. Maayan R. Delayahu Y. Roska P. (2018). A DNA methylation signature of addiction in T cells and its reversal with DHEA intervention. Front. Mol. Neurosci. 11, 322. 10.3389/fnmol.2018.00322 30250424 PMC6139343

[B28] LeMoult J. Humphreys K. L. Tracy A. Hoffmeister J. A. Ip E. Gotlib I. H. (2020). Meta-analysis: exposure to early life stress and risk for depression in childhood and adolescence. J. Am. Acad. Child Adolesc. Psychiatry 59, 842–855. 10.1016/j.jaac.2019.10.011 31676392 PMC11826385

[B29] Li M. D'Arcy C. Li X. Zhang T. Joober R. Meng X. (2019). do DNA methylation studies tell us about depression? A systematic review. Transl. Psychiatry 9, 68. 10.1038/s41398-019-0412-y 30718449 PMC6362194

[B30] Li Y. Ma Q. Dasgupta C. Halavi S. Hartman R. E. Xiao D.. (2017). Inhibition of DNA methylation in the developing rat brain disrupts sexually dimorphic neurobehavioral phenotypes in adulthood. Mol. Neurobiol. 54, 3988–3999. 10.1007/s12035-016-9957-4 27311770 PMC5161729

[B31] Lippard E. T. C. Nemeroff C. B. (2020). The devastating clinical consequences of child abuse and neglect: increased disease vulnerability and poor treatment response in mood disorders. Am. J. Psychiatry 177, 20–36. 10.1176/appi.ajp.2019.19010020 31537091 PMC6939135

[B32] Liu D. Diorio J. Tannenbaum B. Caldji C. Francis D. Freedman A. (1997). Maternal care, hippocampal glucocorticoid receptors, and hypothalamic-pituitary-adrenal responses to stress. Science 277, 1659–1662. 10.1126/science.277.5332.1659 9287218

[B33] Liu X. S. Wu H. Ji X. Stelzer Y. Wu Y. Czauderna S.. (2016). Editing DNA methylation in the mammalian genome. Cell 167, 233.e17–247.e17. 10.1016/j.cell.08.056 27662091 PMC5062609

[B34] McEwen L. M. O'Donnell K. J. McGill M. G. Edgar R. D. Jones M. J. MacIsaac J. L.. (2020). The PedBE clock accurately estimates DNA methylation age in pediatric buccal cells. Proc. Natl. Acad. Sci. U.S.A. 117, 23329–23335. 10.1073/pnas.1820843116 31611402 PMC7519312

[B35] McGowan P. O. Sasaki A. D'Alessio A. C. Dymov S. Labonte B. Szyf M.. (2009). Epigenetic regulation of the glucocorticoid receptor in human brain associates with childhood abuse. Nat. Neurosci. 12, 342–348. 10.1038/nn.2270 19234457 PMC2944040

[B36] Melas P. A. Wei Y. Wong C. C. Sjoholm L. K. Aberg E. Mill J. (2013). Genetic and epigenetic associations of MAOA and NR3C1 with depression and childhood adversities. Int. J. Neuropsychopharmacol. 16, 1513–1528. 10.1017/S1461145713000102 23449091

[B37] Morris M. J. Na E. S. Autry A. E. Monteggia L. M. (2016). Impact of DNMT1 and DNMT3a forebrain knockout on depressive- and anxiety like behavior in mice. Neurobiol. Learn. Mem. 135, 139–145. 10.1016/j.nlm.2016.08.012. 27545441 PMC5050143

[B38] Nagy C. Suderman M. Yang J. Szyf M. Mechawar N. Ernst C. (2015). Astrocytic abnormalities and global DNA methylation patterns in depression and suicide. Mol. Psychiatry. 20, 320–328. 10.1038/mp.2014.21 24662927 PMC5293540

[B39] Okada S. Morinobu S. Fuchikami M. Segawa M. Yokomaku K. Kataoka T. (2014). The potential of SLC6A4 gene methylation analysis for the diagnosis and treatment of major depression. J. Psychiatr. Res. 53, 47–53. 10.1016/j.jpsychires.2014.02.002. 24657235

[B40] Payne J. L. Osborne L. M. Cox O. Kelly J. Meilman S. Jones I. (2020). DNA methylation biomarkers prospectively predict both antenatal and postpartum depression. Psychiatry Res. 285, 112711. 10.1016/j.psychres.2019.112711 31843207 PMC7702696

[B41] Rush A. J. Trivedi M. H. Wisniewski S. R. Nierenberg A. A. Stewart J. W. Warden D. (2006). Acute and longer-term outcomes in depressed outpatients requiring one or several treatment steps: a STAR^*^D report. Am. J. Psychiatry 163, 1905–1917. 10.1176/ajp.163.11.1905 17074942

[B42] Sales A. J. Biojone C. Terceti M. S. Guimaraes F. S. Gomes M. V. Joca S. R. (2011). Antidepressant-like effect induced by systemic and intra-hippocampal administration of DNA methylation inhibitors. Br. J. Pharmacol. 164, 1711–1721. 10.1111/j.1476-5381.2011.01489.x 21585346 PMC3230817

[B43] Sarris J. Murphy J. Mischoulon D. Papakostas G. I. Fava M. Berk M. (2016). Adjunctive Nutraceuticals for depression: a systematic review and meta-analyses. Am. J. Psychiatry 173, 575–587. 10.1176/appi.ajp.2016.15091228 27113121

[B44] Saunderson E. A. Spiers H. Mifsud K. R. Gutierrez-Mecinas M. Trollope A. F. Shaikh A. (2016). Stress-induced gene expression and behavior are controlled by DNA methylation and methyl donor availability in the dentate gyrus. Proc. Natl. Acad. Sci. U.S.A. 113, 4830–4835. 10.1073/pnas.1524857113 27078100 PMC4855538

[B45] Sharma A. Gerbarg P. Bottiglieri T. Massoumi L. Carpenter L. L. Lavretsky H. (2017). S-Adenosylmethionine (SAMe) for neuropsychiatric disorders: a clinician-oriented review of research. J. Clin. Psychiatry 78, e656–e667. 10.4088/JCP.16r11113 28682528 PMC5501081

[B46] Story Jovanova O. Nedeljkovic I. Spieler D. Walker R. M. Liu C. Luciano M. (2018). DNA methylation signatures of depressive symptoms in middle-aged and elderly persons: meta-analysis of multiethnic epigenome-wide studies. JAMA Psychiatry 75, 949–959. 10.1001/jamapsychiatry.2018.1725 29998287 PMC6142917

[B47] Suderman M. McGowan P. O. Sasaki A. Huang T. C. Hallett M. T. Meaney M. J.. (2012). Conserved epigenetic sensitivity to early life experience in the rat and human hippocampus. Proc. Natl. Acad. Sci. U.S.A. 2, 17266–17272. 10.1073/pnas.1121260109 23045659 PMC3477392

[B48] Unternaehrer E. Luers P. Mill J. Dempster E. Meyer A. H. Staehli S. (2012). Dynamic changes in DNA methylation of stress-associated genes (OXTR, BDNF ) after acute psychosocial stress. Transl. Psychiatry 2, e150. 10.1038/tp.2012.77 22892716 PMC3432191

[B49] Ursini G. Bollati V. Fazio L. Porcelli A. Iacovelli L. Catalani A. (2011). Stress-related methylation of the catechol-O-methyltransferase Val 158 allele predicts human prefrontal cognition and activity. J. Neurosci. 31, 6692–6698. 10.1523/JNEUROSCI.6631-10.2011 21543598 PMC6632869

[B50] Vijayendran M. Beach S. R. Plume J. M. Brody G. H. Philibert R. A. (2012). Effects of genotype and child abuse on DNA methylation and gene expression at the serotonin transporter. Front. Psychiatry 3, 55. 10.3389/fpsyt.2012.00055 22707942 PMC3374463

[B51] Vojta A. Dobrinic P. Tadic V. Bockor L. Korac P. Julg B. (2016). Repurposing the CRISPR-Cas9 system for targeted DNA methylation. Nucleic Acids Res. 44, 5615–5628. 10.1093/nar/gkw159 26969735 PMC4937303

[B52] Weaver I. C. Cervoni N. Champagne F. A. D'Alessio A. C. Sharma S. Seckl J. R. (2004). Epigenetic programming by maternal behavior. Nat. Neurosci. 7, 847–854. 10.1038/nn1276 15220929

[B53] Weaver I. C. Champagne F. A. Brown S. E. Dymov S. Sharma S. Meaney M. J. (2005). Reversal of maternal programming of stress responses in adult offspring through methyl supplementation: altering epigenetic marking later in life. J. Neurosci. 25, 11045–11054. 10.1523/JNEUROSCI.3652-05.2005 16306417 PMC6725868

[B54] Weaver I. C. Meaney M. J. Szyf M. (2006). Maternal care effects on the hippocampal transcriptome and anxiety-mediated behaviors in the offspring that are reversible in adulthood. Proc. Natl. Acad. Sci. U.S.A. 103, 3480–3485. 10.1073/pnas.0507526103 16484373 PMC1413873

[B55] Xu S. J. Heller E. A. (2019). Recent advances in neuroepigenetic editing. Curr. Opin. Neurobiol. 59, 26–33. 10.1016/j.conb.2019.03.010. 31015104 PMC12826455

[B56] Yamagata H. Ogihara H. Matsuo K. Uchida S. Kobayashi A. Seki T. (2021). Distinct epigenetic signatures between adult-onset and late-onset depression. Sci. Rep. 11, 2296. 10.1038/s41598-021-81758-8 33504850 PMC7840753

[B57] Zhao J. Goldberg J. Bremner J. D. Vaccarino V. (2013). Association between promoter methylation of serotonin transporter gene and depressive symptoms: a monozygotic twin study. Psychosom. Med. 75, 523–529. 10.1097/PSY.0b013e3182924cf4 23766378 PMC3848698

